# Follow up study of symptomatic human cystic echinococcosis treatment with albendazole and praziquantel, in Uruguay

**DOI:** 10.1186/s12879-024-09539-y

**Published:** 2024-07-25

**Authors:** Daniel Da Rosa, Elisa Figueredo, Michel Rosas, Fernando Goñi

**Affiliations:** 1 Departament of Parasitology and Mycology, Instituto de Higiene, Facultad de Medicina, Universidad de la República - UDELAR, Montevideo, Uruguay; 2 Department of Neurology, New York University Grossman School of Medicine, New York, USA

**Keywords:** Cystic echinococcosis, Medical treatment, Albendazole, Praziquantel, Uruguay

## Abstract

**Background:**

Cystic echinococcosis (CE) is a chronic disease considered a neglected one. Cystic echinococcosis is endemic in Uruguay and the region. Surgery, using various technical approaches, has the potential to safely remove the cyst(s) and lead to a complete cure in a high number of patients with simple forms of CE. However, surgery may be impractical in patients with multiple cysts in several organs, high surgical risk, or in patients with previous multiple surgeries. In these cases, the pharmacological treatment with the benzimidazolic drug Albendazole (ABZ) alone or combined with Praziquantel (PZQ), has been promising as the best choice to achieve improvement or cure.

**Methods:**

In this study, we analyze the results obtained on the anti-parasitic treatment of 43 patients diagnosed with CE between the years 2003 and 2020. Patients were treated before and/or after surgery with ABZ or the combination ABZ/PZQ. The standardize protocol of the anti-parasitic drug treatment before surgery was 7 days, 15 days or 1 month depending on the urgency and availability of the surgical procedure. All cases that involved confirmed locations on lungs underwent immediate surgery with minimal pre-treatment when possible. After surgery, the standardize protocol of anti-parasitic drug treatment consisted of six cycles of 30 days each and resting intervals of 15 days in between. ABZ was used in all cases, administered orally, twice daily, at a total dosage of 15 mg/kg/day, with food high in fat content for improved absorption. The follow up was carried out according to WHO-IWGE guidelines for 5 years.

**Results:**

Of the 43 patients fourteen were ≤ 15 years of age and had a differentiated pre-surgical treatment. From the ≥ 16 years of age, 36 completed the treatments and the 5 years follow up. Four patients changed geographical locations, without a forwarding contact, after the post-surgery treatment. No patient died during the study. Of the 36 patients that completed the study, 32 were treated only with ABZ; 93.75% achieved treatment success as determined by improvement or cure, and 6.25% treatment failure determined by no change or worsening. The last four patients received the ABZ/PZQ combination therapy and achieved 100% treatment success.

**Conclusion:**

The pharmacological treatment resulted in a good option not only as palliative but also as potentially curative. The main relevance of its use was in cases with previous multiple surgeries or surgeries with potential life-threatening complications due to the number and location of cysts and concurrent comorbidities. A follow-up of at least 5 years would be recommended to assure remission and control of the transmission. More randomized trials are needed to provide clear clinical evidence of different pharmacological treatments for CE.

## Introduction

Cystic echinococcosis (CE) is a zoonosis that in humans is caused by the metacestodes of the *Echinococcus granulosus*, and transmitted by the adult stage liberated from the intestines of dogs and others carnivores. Cystic echinococcosis is a major zoonosis endemic to many regions of the world including all South America. In Uruguay, it is considered a serious public health problem [1]. The prevalence in humans with a high risk of exposure in the rural areas of Uruguay, is 3.6 per 1.000 inhabitants [2], albeit the much lower incidence (0.07%) among schoolchildren. Nevertheless, there are about 70 new cases reported each year. The same rate of transmission was seen in a few cases involving minors of 15 years of age or less [3].

All the different surgical approaches have the potential to eliminate cysts, conducting in many cases to a complete cure. However, in later years specific anti-parasitic drug treatments have gone from the alternative use in inoperable cases, to a powerful tool for improvement before and after surgical procedures. Of increased value would be the use in patients with multi-cysts in different locations, previous multiple surgeries, and patients with co-morbidities or high surgical risk [4, 5].

The pharmacological treatments included the benzimidazole drug derivative Albendazole (ABZ) or the Praziquantel (PZQ), either alone or in combination [6–10].

To assess the effects of the pharmacological therapies different imaging techniques are recommended, including Ultra Sonography (US), computed tomography (CT), magnetic resonance imaging (MRI) and X-rays (XR). They all should help determine the location, number, size and status of the cysts. However, when combined with improved biochemical techniques performed in biological fluids; the enhanced monitoring of the effects provides an earlier and better insight of the occurring changes in the cysts during different treatments, resulting in potential clinical improvements [11, 12].

Immunological assessment of the CE status using specific class and subclass antibody responses, circulating antigens and immune-complexes have been reported in several studies [13–15]. Such laboratory tests do provide important additional and supporting clinical data for patients requiring long-term treatments of severe CE.

In this work, we present the experience of the detection, treatment and individual 5 years follow-up during the period of 2003 to 2020. All cases involved severe, partially inoperable or secondary disseminated echinococcosis, treated with extended chemotherapy using Albendazole and Praziquantel. Different imaging and biological methods were used to determine the remission of the disease during follow up.

## Methods

This is a cohort prospective observational study conducted between January 2003 and December 2020 in the Departamento de Parasitología y Micología, Facultad de Medicina, UDELAR, Montevideo, Uruguay.

### Recruitment

Patients were recruited from January 2003 to October 2020, referred by several hospitals belonging to the Sistema Nacional Integrado de Salud (Health Integrated National System). This presentation is focus on the patients that completed or were part of the 5 years follow-up post-treatment at October 2020; and includes the ones that received anti-parasitic drug treatment before surgery.

### Definition of cases

#### Suspected case

Irrespective of the age at the presentation, all patients with the presence of a cyst mass in different organs detected by imaging studies or derived from the clinical and epidemiological link to the disease; i.e., dyspnoea, pain due to abnormal compressing mass in abdomen or other location, history of contact with canines potentially from infected areas or the confirmation of in-living relatives with Cystic Echinococcosis.

#### Probable case

Same considerations as in Suspected Cases but with a positive serological test. A negative serology cannot exclude the diagnosis of cystic echinococcosis.

#### Confirmed case

Same considerations as in the suspected or probable cases but with a biopsed cyst after surgery showing on microscopy either protoscolices, parasite hooks, or remains of the typical membranes.

#### Criteria for inclusion in the cohort

(a) patients with Cystic Echinococcosis diagnosis, based on clinical symptoms, morphological features detected by imaging techniques (RX, US, CT, MR) and immunologic tests comerciales: indirect hemagglutination-HAI (Cellognost Echinococcosis, Siemens, Germany) and indirect immunofluorescence-IFI (Biocientica SA, BA, Argentina), (b) cysts of 7 cm or less or equal size remnants of post-surgery, high surgical risk or cysts in multiple locations, (c) anti-parasitic drug treatment before surgery or (d) stages CE1, CE2 o CE3a o CE3b, as defined by WHO-IWGE.

#### Surgical treatment

Patients with low or moderate surgical risk and cysts larger than 7 cm, were subjected to surgery. Our protocols required mandatory surgery for any cysts with pulmonary location.

### Pharmacological anti-parasitic treatment protocol and follow-up

The Ethics Committee of the School of Medicine UDELAR approved the protocol in the year 2000.

Before surgery, the standardize anti-parasitic drug treatments were either 7 days, 15 days or 1 month, depending on the evaluated risk for a safe period before the surgical procedure.

After surgery, we used a discontinuous anti-parasitic drug treatment of six 30 days cycles with 15 days resting intervals in between cycles.

The only exemptions were patients with cysts in lung locations. The surgery was performed immediately with minimal 7 days pre-surgery anti-parasitic treatment when possible. All lung cases received anti-parasitic drug treatment post-surgery.

The drug was ABZ in all cases, administered orally, twice daily, at a total dosage of 15 mg/kg/day, with food high in fat content for improved absorption [16, 17].

When possible, including all the underdeveloped suburban and rural areas of the Country; the biological fluid tests included blood count and complete functional hepatic to visualize potential side effects of the ABZ and evaluation of the general clinical state of every patient. Minimally, we requested hematological determinations to assess leukopenia (≤ 5000 leukocytes) and hypertransaminasemia of ≤ 150U/L, a number over three times the GOT and GPT normal values according to age and sex. In cases of verified ABZ toxicity, the dosage was immediately lowered to 7,5 mg / kg / day and the blood count and functional hepatic test run weekly until the values normalized. In case of continuing hematological alterations and/or hypertransaminasemia; ABZ had to be suspended until all the abnormal values disappeared. Only then, ABZ delivery was resumed at 7.5 mg / kg / day, associated with PZQ at a dosage of 40 mg / kg / day, allowing to continue the follow-up with frequent monitoring of blood and liver parameters.

### Treatment efficacy and follow-up

In abdominal cases, a follow-up ultrasound was performed every 3 months in the first year and a CT one year after finishing treatment. Subsequent control was performed with annual CT. Priority for the imaging studies was given to the same professional who produced the original results; using the same conditions to consider the images comparative in the follow-up. The images were analyzed to determine the size and status of individual cysts (standard classification of WHO-IWGE) [7, 8].

The treatment-effectiveness is defined considering imaging evolution five years after the end of the initial pharmacological therapy, and the status of any remaining cysts determined by the standard classification of WHO-IWGE.

According to our data, individual patients and cysts were classified as achieving success –determined by cure or marked improvement-, or no success –determined by no change or worsening-. Cure was defined as disappearance of the cyst(s) determined by TC parameters; marked improvement was defined as ≥ 25% reduction of the cyst size; both definitions required transition to inactive phases (CE4, CE5) in follow-up evaluation.

#### Ethics statement

 All patients gave their written informed consent for pharmacological treatment and follow-up. All children under the age of 18 required written consent from the parents or legal guardians. The Ethics Committee of the School of Medicine, UDELAR, approved the study.

#### Statistical analysis

 All the patient clinical and laboratory data was analyzed by Epi Info 2000 (Center for Disease Control, Atlanta, Georgia, USA). Data was described as mean value ± SD or frequency and percentage when appropriate.

## Results

Forty-three patients were newly diagnosed with cystic echinococcosis between January 2003 and December 2020.

All patients presented clinical symptoms and epidemiological link to all geographical areas of the country.

Of the 43 patients: 14 were children under 15 years of age and 29 were over 16 years of age;20 (46%) were women and 23 (54%) were males (Table 1). They all were referred from suburban and rural areas of different geographical Departments of the Country (Cerro Largo, Canelones, Colonia, Rivera, Salto).

**Table 1:** Main epidemiological and clinical data from 43 patients included in this study

Age	Age in years (Median and range)	47,5 (6–75)
	≤ 15 years	14 (33%)
	≥ 16 years	29 (67%)
**Sex**	Women	20 (~ 46%)
	Men	23 (~ 54%)
**Location of cysts**	Liver	26
	Lung	10
	Heart	1
	Brain	1
	Splenic	1
	Kidney	1
	Muscle	3
	Multiple organs	14
**Serology (HAI and IFI)**	Positive	37 (86%)
	Negative	6 (14%)
CE Stage *	CE1	24
	CE2	19
	CE3a	7
**Complications related to hydatic cysts**	Compression and pain in adjacent structures	39
	Hydatic vomic of ruptured cysts	2
	Migration intact to Different locations	2

*Seven patients presented multiple cysts with independent different stages in the different locations

The most frequently affected organ was the liver, accounting for 26 cases (58%), followed by the lung in 10 cases (23%) and 7 in other organs. In 14 cases (19%), individual echinococcal cysts were observed in various different organs. (Table 1)

In patients with images compatible with known cystic echinococcosis, HAI and IFI was always performed: 37 (86%) patients showed positive results, and 6 (14%) patients resulted negative for both studies.

The most commonly observed cystic stages according to WHO-IWGE classification, corresponded to stages CE1 in 24 cases, CE2 in 19 cases, CE3a in 7 cases and CE3b in 2 cases. Seven of the 14 cases confirmed with multi-organ cysts locations presented different CE stages, and were recorded as different stages in the same patient (Table 1).

All patients reported abnormal symptoms, its presentation depending on the localization, size and integrity of the cysts. Thirty-nine patients (90%) reported pain and discomfort due to compression of anatomical structures adjacent to the cysts. Two cases presented hydatic vomica product of the opening or rupture of cysts located in lungs or respiratory tract; and two cases diffused abdominal symptoms due to migration to different locations in the pelvic cavity of cysts released from the rupture of the Glisson capsule in the surface of the lower segment of the liver.

Fourteen patients with multiple cysts larger than 7 cm in different locations presented low surgical risk due to their topography and accessibility. The cysts were surgically removed after the first examination with anti-parasitic drug treatment when possible.

Fourteen cases were children under 15 years of age; eight with a single cyst (6 liver and 2 lung) received only pre-surgical anti-parasitic drug treatment: four (2 lung and 2 liver) for 7 days, two (liver)for 15 days and two (liver) 30 days. The remaining six patients underwent surgery without prior anti-parasitic drug treatment. These six children received post-treatment according to the protocol; 2 completed the 5 years follow up, and 4 are still under evaluation (Table 2).

**Table 2:** Anti-parasitic drug treatment of patients with complete follow-up (*n* = 36)

Drug and Time of Anti-parasitic Treatment	Number of patients (*n*)	Time in weeks	%
Total treated	36		100
ABZ only	32		88
Combined ABZ-PZQ	4		12
Not treated before surgery	11		22
≤ 15 years; Treated before surgery	8		22
≥ 16 years; Treated before surgery	18		78
≤ 15 years; Treatment after-surgery	6		17
≥ 16 years; Treatment after-surgery	30		83
Post treatment follow up		240	
Adverse effects	3		8.3
None registered	32		89
Leucopenia	1		2.8
Alopecia	1		2.8
Hipertranssaminasemia	2		5.6

For the 29 patients over 16 years of age; 18 received pre-surgical anti-parasitic drug treatment: 2 (lung location) for 7 days; 7 for 15 days and 8 for 30 days. The efficacy of the pre-surgical treatment was determined by the 2% eosin staining viability test. The number of non-viable protoscolices decreased substantially depending on the length of the anti-parasitic treatment (Table 3). The remaining 11 patients with multiple cysts location or cysts over 7 cm in size, underwent surgery without previous anti-parasitic drug treatment. All 29 patients received post-surgical anti-parasitic drug treatment.

**Table 3:** Viability of protoscolices vs. days of treatment with ABZ before surgery (*n* = 25)

Number of patients (*n*)	Days of treatment	Protoscolices	
		% viable	% non-viable
6	7	57	43
9	15	33	67
10	30	3	97

From the 43 patients included in the study 36 completed the treatments and the 5 year follow up. Seven patients changed geographical location without forwarding contact information and dropped out of the study before completing the treatment or the 5 years follow up.

After thorough risk assessment, 5 of the 10 cases with pulmonary location received a maximum of 7 days pre-surgery albendazole anti-parasitic drug treatment. Four cases underwent surgery immediately without pre-treatment. All nine cases with lung location completed the post-surgical anti-parasitic drug treatment. The remaining case presenting extreme complexity of multiple small pulmonary cysts and vomic had to be treated exclusively with albendazole according to the post-surgery anti-parasitic drug treatment and showed a successful cure with disappearance of the cysts and a clean follow up (Table 2). In all cases, post-surgical treatment continued for 6 cycles over 9 months. Follow-up of the treatment 240 weeks (Table 2).

Of the 16 who presented remaining cysts after surgery and treatment; 10 (62.5%) progressed to inactive stages and were classified as improvement; 4 cases (25%) evolved from active stages to a completely inactive stage and were considered cured. One case (6.25%), an elderly patient with high surgical risk, remained in a transition stage during treatment, and is still being evaluated by “Watch and Wait Approach” annual imaging [4] (Table 4). An interesting case involves the remaining patient. Seven years prior to the begining of this study presented a single large cyst (≥ 20 cm) located in the lung with an episode of vomic; underwent immediate surgery and was later treated with Nebendazole. He consulted for dyspnea and was included in the study after observing multiple inoperable small cysts (~ 2 cm) only located in the lung. The patient received the anti-parasitic discontinuous drug (ABZ) for the six 30 days cycles over a period of 9 months. At the end of the treatment, CT imaging could not detect any remaining cyst and the patient remained free of the disease for the reminder of the 5 years follow up (Table 4).

**Table 4:** Outcome measures of patient with pharmacological treatment (*n* = 36)

Drug	*N*° of cases	Stage WHO-IWGE Before treatment		Clinical evolution		Stage WHO-IWGE After treatment		%	
ABZ	16	CE1, CE2 CE3a, CE3b		Successful surgery (*n* = 16)		no recurrences were observed		100	
		CE1, CE2, CE3b	Improvement (*n* = 10)		CE 3a, CE4 CE5		62,5		
ABZ	16	CE1		Cured (*n* = 1)		Pulmonary case. Cysts disappeared		6.25	
		CE2, CE3a		Cured (*n* = 4)		CE5		25	
		CE3a		Equal (*n* = 1)		CE3a		6,25	
ABZ and PZQ	4	CE2		Improvement (*n* = 2)		CE3a, C4		50	
		CE1		Cured (*n* = 2)		CE5		50	

Adverse effects were observed in four cases: one case of a child under 10 years of age with hypertransaminaemia; and three cases of patients over 16 years of age: two with leukopenia and one with alopecia. The four were treated with the combination of albendazole and praziquantel and achieved either improvement or cure (Table 4).

## Discussion

This study explores the feasibility and safety of the controlled discontinuous use of ABZ alone or in combination with PZQ, to treat before and after surgery hydatid cases of advanced active stages classified according to the WHO-IWGE system. The results and the follow-up are encouraging with most of the patients progressing to inactive stages, or in some cases achieving complete remission after elimination of small cysts. In our experience, this pharmacological treatment can be deemed successful and suitable to be used in the future. The expectation is focus in lowering the size of the cysts, progressing to inactive stages and diminishing the crippling symptoms associated with the cyst(s) mass in different locations.

The recommendation of experts in the last 20 years is to change discontinuous treatments to continuous treatments, for at least 2 months in disseminated cases and in immunocompromised patients [18]. Studies carried out by Horton in a 12-year study of discontinuous treatment led to the conclusion that the efficacy of the treatment was 71%, but the distribution by stage was not taken into account. At the same time, studies of 3 to 6 months of continuous treatment were carried out, in which 82% of cysts with degenerative changes were obtained, recently it was observed that in active and transition cysts a success of 34% [16, 19]. However, to date there is no complete decision on which of the treatment options is the most effective.

In our study, we followed the triple diagnosis recommended by WHO-IWGE; combining a clinical diagnosis, suspicious imaging and immunological confirmation. The later used to confirm patients with no previous diagnosis of CE. These cases usually came to the consult at the Health Centers referring pain and “mass sensation” in the right hypochondrium, not necessarily specific of a particular disease. Subsequent imaging were mostly diagnostic and suggested cysts on active stages. However, 14% of cases with positive images presented negative immunological results for Echinococcal antigens, albeit not excluding a positive diagnosis; and requiring more advanced imaging techniques, MRI, or when possible surgically removing the cyst to confirm or discard CE. Nevertheless, these cases received preventive treatment and a 2 years follow-up, even if on pathological examination the nature of the cyst was not the product of any parasitic infection.

The immunological studies in biological fluids have had promising developments that might help future follow-up of patients undergoing pharmacological treatment of CE. The best candidate is the 2B2t recombinant antigen, an excellent tool to monitor the treatment of unilocular hydatid disease, and determine ongoing risk or cure [20].

More recently, Petrone and collaborators have used a combination of humoral markers; AgB-specific IL-1ra, IL-6, IL-8, FGF, G-CFS, IFN-γ, MIP-1α, IP-10 and MCP-1; to monitor specific activity of the immune system and suggesting a potential role in determining cyst(s) viability linked to the reduction of the markers over time [21]. These type of tests and the ones being developed might be crucial in the follow-up of future patients presenting CE. Nevertheless, given the characteristics and availability of the tests, these studies are today restricted to high complexity laboratories, with sometimes a cost impossible to defray for the Health System in small communities.

In our experience the treatment before surgery is essential to decrease the viability of the protoscolices and the intracystic tension that was observed independently of the surgical technique used. However, if the surgical act resulted in the removal of all cysts, no differences were observed in the follow up between pre- and post-treatment and only post-surgery treatment.

It should be noted that patients with remnant cysts had a cure rate of 31,75% and improvement of 62,5% with ABZ alone; and in the combination of ABZ with PZQ, a 50% improvement and 50% cure. Although the number of cases is low, these data are comparable to other series in the literature [22, 23]. Contrary to other South American Countries [24], in Uruguay the CE presentation is complicated, many times involving cysts at different stages in the same patient. The duration of pharmacological treatment should vary according to the location and size of the cyst(s). We observed an effective therapeutic response in liver or lung cysts when they were less than 7 cm and received an optimal treatment of 6 cycles. Although the size of the cysts in other series is less than 6 cm, the hypothesis is their good response correlate with the maximum dose used (15 mg / kg / day), obtaining a higher bioavailability, intra-cystic concentration and tolerability of the drug [25–28].

In all cases, the follow-up of symptomatic patients was based on the WHO-IWGE recommendations [7, 8]. However, few cases presented difficulties locating migrant patients in different parts of the Country. Our Clinic, a reference for Echinococcosis in Uruguay, prepared medical/chirurgical decentralized teams to act as educators for continuous medical programs in parasitic zoonosis. The fact that epidemiologically the CE presentation in the last 20 years has moved from the rural to the suburban areas has benefited in part the localization of risk cases, but it has complicated the logistics given the socio-economic status of the cities peripheries.

The adverse effects were restricted to increased liver enzymes, leukopenia and hair loss; as expected for treatments with benzimidazoles [23]. We did not observed secondary effects on the ABZ + PZQ combined therapy; probably due to the length and dosage of the treatment combined with the fast and positive outcome observed on these patients. Nevertheless, as encouraging, as these results are, the number of patients was small to show a clear result. Larger cohorts are needed to corroborate these data.

Still, we maintain the need for 5 years follow-up after treatment. During that period, we in this study and others have demonstrated the structural and morphological changes of the cysts and the disappearance of small cysts, mainly pulmonary, in most cases [8, 23, 29, 30].

In sum, we observed the pharmacological treatment is a good option not only as palliative but also as potentially curative. The main relevance of its use was in cases with previous multiple surgeries or surgeries with potential life-threatening complications due to the number and location of cysts and concurrent comorbidities. A follow-up of at least 5 years would be recommended to assure remission and control of the transmission. More randomized trials are needed to provide clear clinical evidence of different pharmacological treatments for CE.

## Data Availability

In the manuscript submitted for publication, all patient personal data or identification are excluded, maintaining total anonymity. The data sets generated and/or analyzed during the current study could be available, maintaining anonymity, from the corresponding author upon reasonable request.

## Declarations

### • WHO International Clinical Trial Registry platform

Assign ISRCTN to trial - standard fee https://www.isrctn.com/ISRCTN78387420.

02/08/2023 ISRCTN78387420 Pre- and post-surgical medical treatment of human cystic echinococcosis.
